# HIV Prevention Continuum Outcomes Following Implementation of a Municipal HIV Self-Testing Program

**DOI:** 10.1007/s10461-025-04842-4

**Published:** 2025-08-14

**Authors:** Cedric H. Bien-Gund, Knashawn Morales, Javontae Williams, Brian Hernandez, Brandon Ptak, Kathleen Brady, Robert Gross

**Affiliations:** 1 Division of Infectious Diseases, Department of Medicine, Perelman School of Medicine, University of Pennsylvania, 3400 Spruce Street, Philadelphia, PA 19104, USA; 2 Department of Medicine, Perelman School of Medicine, University of Pennsylvania, Philadelphia, USA; 3 Leonard Davis Institute of Health Economics, University of Pennsylvania, Philadelphia, USA; 4 Center for Clinical Epidemiology and Biostatistics, Perelman School of Medicine, University of Pennsylvania, Philadelphia, USA; 5 Division of Biostatistics, Department of Biostatistics, Epidemiology, and Informatics, Perelman School of Medicine, University of Pennsylvania, Philadelphia, USA; 6 Division of HIV Health, Philadelphia Department of Public Health, Philadelphia, USA

**Keywords:** HIV self-testing, Pre-exposure prophylaxis, Prevention continuum, Public health program

## Abstract

HIV self-testing (HIVST) must lead to engagement in the HIV status neutral continuum to maximize its benefits. The objective of this research was to determine the reach of a public health HIVST program, characterize the HIV prevention continuum following self-testing, and identify correlates associated with obtaining post-test care and discussing PrEP. We prospectively recruited individuals who obtained an HIVST through a municipal program in Philadelphia, a metropolitan area with high burden of HIV. We examined factors associated with seeing a provider after self-testing, and among those who saw a provider, factors associated with discussing PrEP. Between October 2022 and March 2024, 282 people met inclusion criteria. Men who have sex with men (MSM) comprised 28% of the study sample, 22% identified as Black cis-gender women, and 22% reported no prior HIV test. At one-month follow-up, 53% of respondents with HIV-negative/unknown status saw a provider, but less than a quarter of those discussed PrEP. Black individuals were more than twice as likely to see a provider compared with White individuals. Among those who saw a provider, MSM and Latinx/e individuals were more likely to discuss PrEP, while those assigned female at birth were less likely to discuss PrEP. Implementation of a municipal HIVST program can advance health equity by reaching priority populations, including previously untested persons. Although over half of participants saw a provider after self-testing, very few discussed or initiated PrEP. Interventions to promote linkage to care and PrEP uptake are needed to maximize the impact of HIVST.

## Introduction

Widespread HIV testing is the critical first step in the HIV status neutral care continuum, followed by pre-exposure prophylaxis (PrEP) initiation among individuals at risk of HIV acquisition [1-3]. Unfortunately, efforts to end the HIV epidemic in the US have been hindered by longstanding inequitable access to HIV services, including HIV testing and PrEP, particularly among sexual and gender minority populations and communities of color [4, 5]. Despite a 12% decline in new HIV diagnoses between 2018 and 2022, health inequities have contributed to the failure to reduce new HIV infections by 75% by 2025, a major goal of the national Ending the HIV Epidemic (EHE) initiative [1, 6].

One strategy to increase test uptake is through the use of HIV self-tests (HIVST). In the US, the OraQuick HIVST (OraSure Technologies, Bethlehem, PA) is the only HIV test authorized by the FDA for self-testing [7]. The test detects HIV-1 and HIV-2 antibodies in oral fluid, with test results available within 20 min. HIVST has been shown to be effective in identifying new HIV infections and increasing test use, particularly among populations not reached through conventional testing approaches [8, 9]. In a randomized controlled trial evaluating internet distribution of HIVST among men who have sex with men (MSM), provision of HIVST identified more than twice as many new HIV infections than providing test information alone, and MSM who had never tested before were more likely to self-test than obtain a conventional test [8].

However, PrEP uptake and engagement in the HIV prevention continuum are necessary in order for the benefits of HIVST to be maximized among those who test negative for HIV and are at risk of infection. Unfortunately, prior studies have found PrEP linkage and uptake to be extremely low among priority populations accessing care [10, 11]. In a recent HIVST demonstration project in the US that focused on MSM, only 8% of self-testers accessed PrEP after self-testing [12]. Similarly, a global meta-analysis of nine HIVST studies found that just 9% accessed PrEP after self-testing [13]. However, prior HIVST studies have largely focused on PrEP awareness and use, without evaluating the multiple steps of the prevention continuum, such as seeing a health care provider, discussing PrEP, and initiating PrEP [10, 14]. Furthermore, little is known about the impact of public health HIVST programs in reaching priority populations beyond MSM [8, 9, 15-17]. Therefore, this study had the following objectives: (1) determine the capability of a municipal HIVST program in reaching priority populations, (2) characterize the HIV prevention continuum among HIV self-testers, and (3) identify correlates associated with seeing a provider after self-testing and discussing PrEP.

## Methods

### Study Setting and Design

We conducted a prospective cohort study of individuals obtaining HIV self-testing through a municipal HIVST program offered by the Philadelphia Department of Public Health (PDPH). Philadelphia is a racially diverse city of 1.6 million, designated a priority jurisdiction by the national EHE initiative with over 20,000 people living with HIV, about 10% of whom have not yet been diagnosed [18]. The HIVST program, supported by the national EHE initiative, provides OraQuick HIVST kits free of charge through online mail-order through a municipal HIV and sexual health web-site (phillykeeponloving.com) and through in-person distribution at community-based organizations and health centers throughout Philadelphia. The online platform also includes links to additional sexual health care, including bacterial sexually transmitted infection (STI) testing, emergency non-occupational post-exposure prophylaxis, and PrEP. Test kits also included information on where to obtain a confirmatory test and ART if the test returned positive, or PrEP and prevention care if the test returned negative (Supplemental File 1).

To receive a test kit and be eligible for the study, individuals needed to (1) be at least 17 years of age, (2) not receive a test kit within the past 90 days, and (3) reside in Philadelphia County. HIVST kits included a QR code and a link to an online survey to be completed after self-testing. We selected this age minimum because the OraQuick HIVST was approved only for individuals aged 17 and older at the time of the study. After clicking on the study link, participants completed a self-guided screening form, and if eligible, they completed an online informed consent form. Participants could opt out or end the survey at any time and were provided with a study contact number and email. A follow-up survey was automatically sent via e-mail 30 days after the baseline survey was completed. Given the sensitive nature of the study, e-mail communications did not mention HIV or sexual health. Participants were compensated $15 for completing each of the surveys. The study team reviewed all surveys for authenticity and duplication.

We obtained a waiver of parental consent for minors aged 17, who under Pennsylvania law and in keeping with local IRB guidance, are considered “mature minors” and are able to consent for themselves for research related to sexual health decision making. This study was reviewed and approved by the PDPH Institutional Review Board on June 13, 2022 (Protocol #2022-26).

### Study Measures

We obtained socio-demographic information including age, sex at birth, gender identity, race, ethnicity, and sexual orientation (Supplemental File 2). We defined MSM as individuals assigned male sex at birth and reporting prior anal sex with a man, including those who indicated transgender and nonbinary gender identities. We combined race and ethnicity measures into a single variable: Black, non-Latinx/e; White, non-Latinx/e; Latinx/e, any race; and other race/multi-racial, non-Latinx/e.

Survey measures included HIVST results, where the test was obtained, prior HIV testing, sexual history, substance use, and medical history including prior PrEP use. These measures were adapted from the National Behavioral HIV Surveillance survey, MSM cycle [19]. If participants indicated a positive test result, we contacted them by phone to confirm the test results and offer post-test linkage to care.

We developed a conceptual framework based on the adapted Integrated Behavioral Model (IBM) [20] (Supplemental File 3). We selected covariates and built separate models including the covariate of interest and con-founders [21], based on our literature review [22-25]. The IBM model holds that behavioral intentions, driven by individual attitudes, norms, and self-efficacy, are the strongest predictor of health behaviors such as seeking a health provider or taking PrEP [20, 26]. Our survey included measures on PrEP intentions, attitudes, norms, and self-efficacy that were previously validated among MSM and transgender women in the US [27]. We adapted survey measures to ask participants about their intentions to talk to a health care provider about their sexual health within the next month, using a Likert scale (1 = definitely will not do, 2 = probably will not do, 3 = probably will do, 4 = definitely will do). If participants indicated HIV-negative or unknown status, we included additional survey items about their intentions to (1) talk about HIV with a provider, (2) discuss PrEP with a provider, (3) seek out more information about PrEP, and (4) get a prescription for PrEP.

We conducted a follow-up survey 30 days after the baseline survey to assess engagement in the HIV prevention continuum after self-testing. The follow-up survey included survey items evaluating steps along the prevention continuum. Items included whether the participant saw a provider, either in-person or virtually, had made an appointment to see a provider, obtained STI testing, discussed sexual health with a provider, discussed PrEP with a provider, obtained PrEP, and initiated PrEP (Supplemental File 4).

### Statistical Analysis

We analyzed descriptive statistics on baseline characteristics of study participants. Among participants who completed the follow-up survey, we used multivariable logistic regression to estimate covariate effects on two outcomes of interest: follow-up with a health care provider after self-testing, and among those who saw a health care provider, whether they discussed PrEP. For the outcome of follow-up with a provider, we controlled for age, race, sex at birth, and insurance. Among participants who saw a provider, we did a subgroup analysis to evaluate factors associated with discussing PrEP, and controlled for age, race, and sex at birth.

## Results

**Fig. 1:**
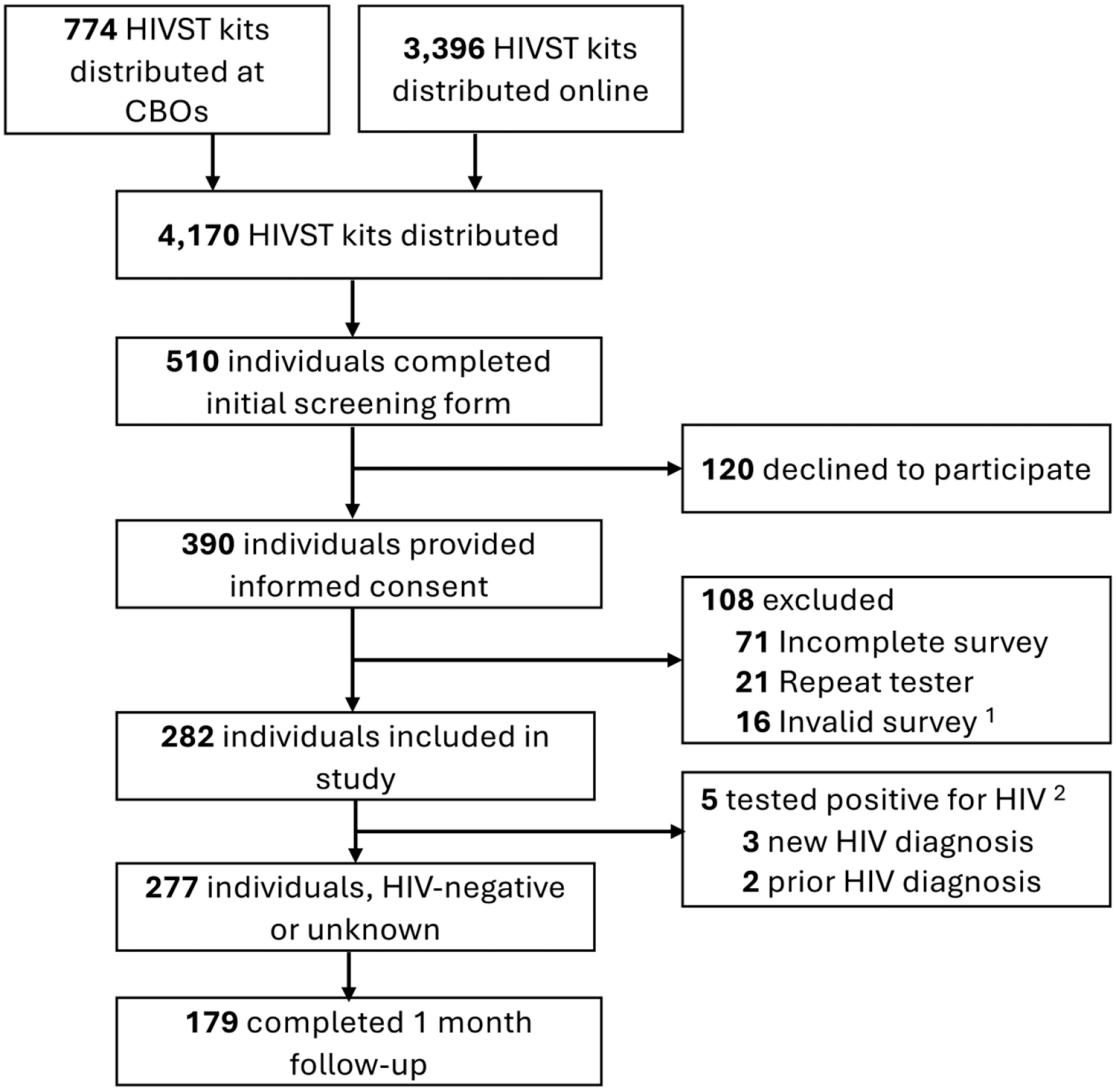
Flow diagram of HIV self-testing cohort ^1^ Surveys deemed invalid if no valid name, phone, email provided. ^2^ Individuals who indicated a positive HIV test were called to confirm whether the diagnosis was new.

Between October 2022 and March 2024, a total of 4170 HIVST kits were distributed online and through community-based organizations (Fig. 1). A total of 510 participants completed the initial screening form during the survey period, of which 390 completed informed consent. After removing duplicate, invalid, and incomplete surveys, a total of 282 individuals completed the online survey and were included in the study. Overall, 210 (74%) obtained a test kit online, 46 (16%) obtained a test kit from a community-based organization or health center, 10 (4%) obtained a test kit from someone they knew, and 17 (6%) obtained a test from somewhere else. A total of 239 (85%) reported a negative HIV test result, 5 (2%) participants reported a positive test result, 35 (12%) had not completed the test yet, and 3 (1%) indicated an indeterminate result. Among those who reported a positive HIV test result, two reported a prior diagnosis, and the other three reported a new HIV diagnosis and all had presented to care and initiated anti-retroviral therapy within one month of self-testing.

Participant demographics and other characteristics are shown in Table 1. The median age was 27, 38% percent identified as Black, 32% identified as White, and 17% identified as Latinx/e (any race). Just over half (52%) of all HIVST individuals identified as cis-gender women, of whom 41% identified as Black. Over a quarter (28%) were MSM, 16% had a bacterial STI diagnosis in the past year, and 2% reported injection drug use in the past year. Sixty-three (22%) participants indicated no prior HIV testing, 15 (5%) were currently on PrEP, 10 (4%) had previously used PrEP and discontinued it, and 77 (27%) were unaware of PrEP.

**Fig. 2:**
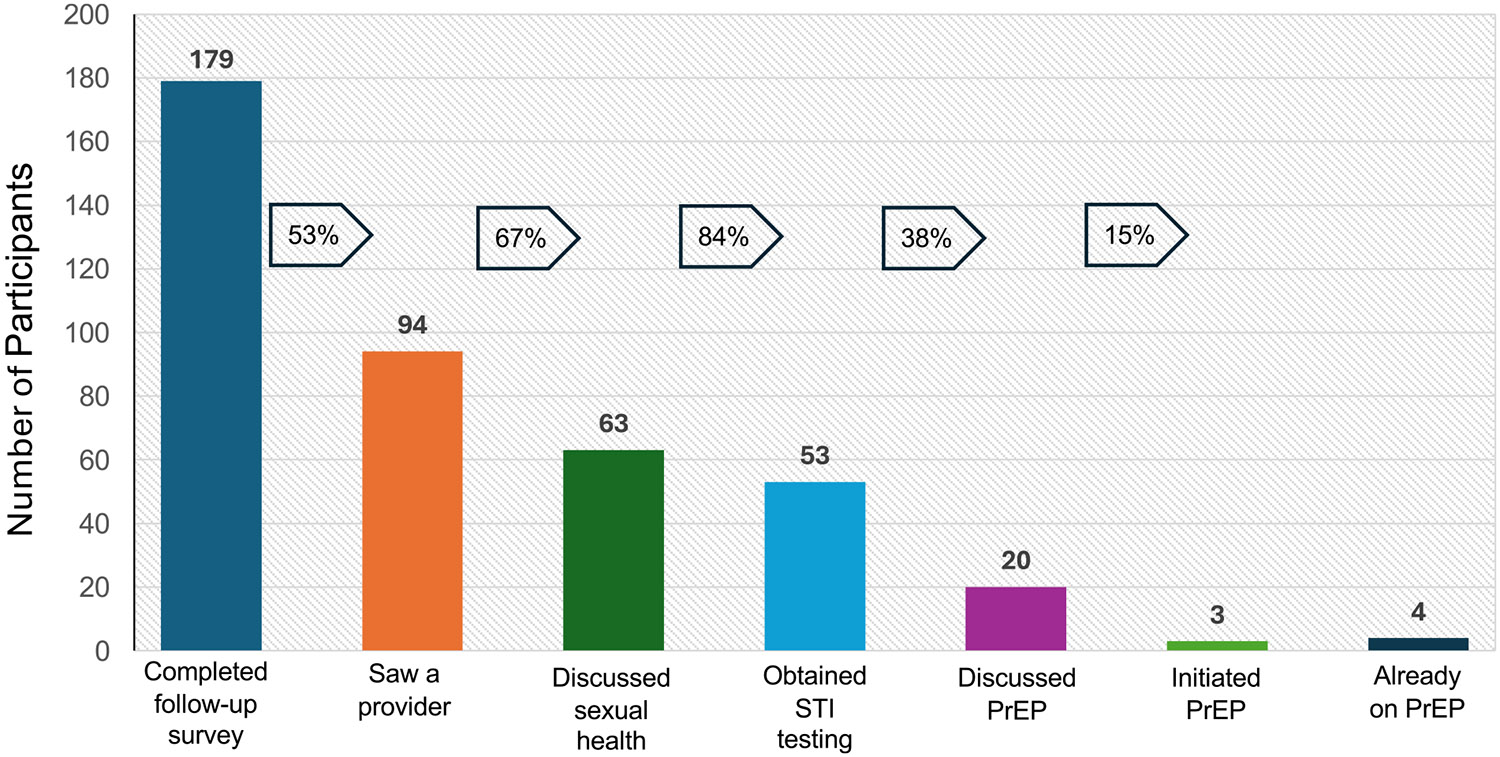
Engagement in the HIV prevention continuum 30 days after self-testing (N = 179)

The follow-up survey evaluated steps along the HIV prevention continuum. Among the 179 participants who completed the follow-up survey and did not test positive for HIV, 94 (53%) had interacted with a medical provider after self-testing (Table 2). Among those who saw a provider, 56 saw a provider in-person and 38 saw a provider virtually. An additional 12 participants had scheduled an appointment but had not seen a provider yet. Among those who saw a medical provider, 63 (67%) discussed sexual health, 53 (56%) obtained additional bacterial STI testing, 20 (21%) discussed PrEP, 4 (4%) were already on PrEP, and 3 (3%) newly initiated PrEP (Fig. 2).

### Factors Correlated with Seeing a Provider and Discussing PrEP

To evaluate engagement in care, we evaluated two outcomes of interest: seeing a provider after self-testing and discussing PrEP. In adjusted analyses, the only factor associated with seeing a provider after self-testing was race, with Black individuals more likely to report seeing a provider compared with White individuals (aOR 2.50, 95% CI 1.16–5.41, p = 0.02) and those who reported other/multi-racial race less likely compared with White individuals (aOR 0.09, 95% CI 0.01–0.74, p = 0.03) (Table 3). While there was a trend toward seeing a provider among those with insurance and with higher intentions to seek care, these associations were not statistically significant.

In examining the next step of the prevention continuum, discussing PrEP with a provider, we found that female individuals were less likely to discuss PrEP compared with male individuals (aOR 0.30, 95% CI 0.10–0.93, p = 0.04) (Table 3). MSM were much more likely to discuss PrEP compared with all other individuals (aOR 18.37, 95% CI 1.89–178.33, p = 0.01), and Latinx/e individuals were also more likely to discuss PrEP compared with White individuals (aOR 6.53, 95% CI 1.23–34.74, p = 0.04).

We did not observe an association between being aware of PrEP with either seeing a provider or discussing PrEP. In addition, seeing a provider or discussing PrEP were not associated with any behavioral covariates we assessed, including STI history, injection drug use, sex with a partner with HIV or unknown status, or reported condom use.

## Discussion

In an urban setting with high prevalence of HIV, a municipal HIVST program successfully reached priority populations, including MSM, transgender and gender diverse persons, Black cis-gender women, and previously untested persons. In contrast with other studies that found low rates of provider follow-up [12, 17], we observed that 53% of individuals interacted with a provider, mostly in-person, within 30 days of follow-up. However, only 21% of those who saw a provider discussed PrEP, with women significantly less likely to discuss PrEP, highlighting significant implementation gaps in the HIV prevention continuum. These findings can inform HIV prevention implementation strategies and design of PrEP linkage interventions.

Our findings demonstrate that a municipal HIVST program can strengthen health equity by reaching priority populations disproportionately affected by the HIV epidemic, including those with no prior HIV testing. Consistent with other studies in the US [8, 12, 17], we found that HIVST successfully reached a large proportion of untested populations, thereby engaging medically disconnected individuals in the HIV prevention continuum for the first time. While other studies on HIVST have focused on MSM [8, 12, 15, 17], our study found that a HIVST program can successfully reach multiple priority populations, including Black cis-gender women, transgender and gender-diverse persons, and a substantial proportion of Black and Latinx/e persons. We also found that only 2% of participants in our study reported injection drug use in the past year, suggesting that additional outreach to people who inject drugs is needed to reach this population. Notably, other studies evaluating HIVST programs have not reported on people who inject drugs in the US [12, 17]. In a prior study of US MSM, we found that HIVST use was associated with those with higher levels of perceived anti-HIV discrimination, demonstrating that HIVST can also reach those with higher levels of stigma [28]. Taken together, these findings should support efforts to expand HIV prevention programs that include HIVST in addressing health equity, particularly given worsening racial and gender disparities in PrEP uptake observed throughout the US [4].

One major concern with HIVST is the lack of follow-up or linkage to care. Prior studies have indicated that follow-up after self-testing is as low as 10–15% [12, 17]. Surprisingly, we observed much higher rates of follow-up, with 53% of respondents interacting with a provider after self-testing, and nearly a third obtaining additional STI testing. One potential reason for this is that the HIVST program was embedded within a “buffet” of sexual health programs offered by the municipal health department, including STI testing and links to PrEP providers, which may have facilitated linkage to care. Additionally, we were encouraged by our finding that Black individuals were more likely to see a provider, given racial inequities in health care and the disproportionate burden of HIV among Black Americans [4, 6, 29].

Despite the higher-than-expected rates of follow-up care and STI testing, we observed low rates of discussing PrEP, highlighting ongoing gaps to link self-testers to PrEP. While systems-level interventions are needed to improve engagement in care after self-testing, our data also suggest that provider-level interventions are needed to promote discussions of sexual health and PrEP. Whereas MSM were much more likely to discuss PrEP once they saw a provider, we found that that women were significantly less likely to discuss PrEP. These findings underscore the need for PrEP interventions for women at risk for HIV, which has remained woefully low [25, 30]. These findings are consistent with calls to increase PrEP discussions with Black cis-gender women, who have been largely excluded from many PrEP implementation efforts [31].

Following PrEP discussions in the prevention continuum, downstream interventions are needed to spur PrEP uptake. A recent randomized controlled trial of HIVST and follow-up counseling found that PrEP counseling did not have an impact on PrEP uptake, highlighting a significant gap between discussing PrEP and actual PrEP uptake [32]. In another study, peer HIVST distribution bundled with PrEP information still resulted in only 2 out of 117 testers initiating PrEP [33]. In addition to PrEP counseling, intensive PrEP navigation or interventions that target other barriers may be needed to facilitate PrEP linkage and uptake [10, 34].

Although there was a trend between intentions to seek care after self-testing and obtaining care at 30 days, this association was not statistically significant. While being assigned male sex at birth and reporting prior sex with men was strongly associated with discussing PrEP with a provider, other behavioral risk factors and behavioral intentions did not predict either outcome of interest: follow-up with a provider or discussing PrEP within 30 days of self-testing. These findings highlight the limited applicability of the IBM framework and behavioral risk factors in predicting outcomes in our study. Prior research has highlighted the complex interplay of behavioral factors, demographic variables, and social determinants of health in predicting HIV acquisition risk [35, 36].

Our study has several limitations. First, our initial survey response rate was low, and respondents to our initial and follow-up survey may not be generalizable to all HIV self-testers. Survey respondents may have had higher motivations to seek medical care. Nonetheless, our follow-up response rate of 63% was significantly higher than other studies which had response rates of 10–20% [12, 17]. Second, we only captured follow-up within 30 days, because we were interested in immediate post-test linkage to care. Participants may have seen a provider or initiated PrEP after that period, which we would not have captured, and we did not assess PrEP adherence or persistence. Third, while we assessed PrEP discussions, we did not assess whether conversations were initiated by the provider or patient, or what type of health care provider the patient saw. However, our observation that a higher percentage of participants discussed sexual health and obtained STI testing than discussed PrEP, strongly suggest that there were missed opportunities for providers to initiate PrEP discussions. Nonetheless, additional qualitative research is needed to understand why PrEP follow-up and discussions of PrEP were so infrequent.

Ending the HIV epidemic will require widespread, equitable uptake of HIV testing and PrEP. Our findings support implementation of a public health HIVST program as an effective strategy to strengthen health equity and expand the reach of testing to key populations. Linkage to care and supporting PrEP uptake after self-testing are critical steps in the HIV prevention continuum that must be addressed. Our analysis suggests that individual-level interventions focused on facilitating HIV self-testers to link to care, as well as provider-level interventions to support PrEP discussions and uptake, are needed to close the large gaps between HIV testing and PrEP uptake.

## Supplementary Material

Supplemental File 3

Supplemental File 2

Supplemental File 4

SUpplemental File 1

**Supplementary Information** The online version contains supplementary material available at https://doi.org/10.1007/s10461-025-04842-4.

## Figures and Tables

**Table 1:** Characteristics of HIV self-testers reached by a HIVST program (N = 282)

Characteristic	N (%)
Age, median (interquartile range)	27 (23, 34)
Sex at birth and gender identity	
Cis-gender man	109 (38.7)
Cis-gender women	147 (52.1)
TG/NB identity, male sex at birth	11 (3.9)
TG/NB identity, female sex at birth	14 (5.0)
Race/Ethnicity	
White, non-Latinx/e	90 (31.9)
Black, non-Latinx/e	107 (37.9)
Latinx/Latine, any race	47 (16.7)
Other race/multi-racial, non-Latinx/e	18 (6.4)
Education	
High school or less	80 (28.4)
Some college	86 (30.5)
College or higher	114 (40.4)
Insurance status	
Uninsured	37 (13.1)
Public insurance	112 (39.7)
Private insurance	120 (42.6)
Source of HIVST kit	
Online	209 (74.1)
CBO or health center	46 (16.3)
Someone they know	10 (3.6)
Somewhere else	17 (6.0)
MSM	79 (28.0)
Sex with a partner with HIV, lifetime	27 (13.4)
Sex with a partner with unknown HIV status, lifetime	202 (72.4)
IDU in past year	5 (1.8)
STI history	
None	152 (53.9)
STI > year ago	72 (25.5)
STI in past year	43 (15.2)
Condom use	
Never	57 (20.2)
Sometimes	183 (64.9)
Always	33 (11.7)
Unaware of PrEP	77 (27.3)
Intention to seek care, Scale 1–4 (SD)^a^	3.03 (0.90)
Intentions to obtain PrEP, Scale 1–4 (SD)^a^	2.31 (1.03)
Prior HIV test	
No prior HIV test	63 (22.3)
Last HIV test > 1 year ago	41 (14.5)
HIV test in past year	178 (63.1)

Rows may not sum to total N due to missing responses

*TG/NB identity* transgender/nonbinary identity, including gender diverse individuals; *CBO* community-based organization; *MSM* men who have sex with men (defined as assigned male sex at birth and prior anal sex with men, including those with transgender/nonbinary gender identities); *IDU* injection drug use; *STI* sexually transmitted infection excluding HIV; *PrEP* pre-exposure prophylaxis; *SD* standard deviation

a Intentions to seek care and obtain PrEP were assigned using a 1–4 scale about likelihood of performing health behaviors in the next month (1 = definitely will not do, 4 = definitely will do)

**Table 2:** Follow-up survey among self-testers after one month (N = 179)

Total N = 179	N (%)		p-value^a^
Characteristic	Saw provider at 30-day follow-up	Did not see a provider at 30-day follow-up	
Total	94 (52.5)	85 (47.5)	
Age, median (interquartile range)	28 (23, 34)	26 (23, 31)	0.34
Sex at birth and gender identity			0.88
Cis-gender man	35 (37.2)	33 (38.8)	
Cis-gender women	49 (52.1)	45 (52.9)	
TG/NB identity, male sex at birth	6 (6.4)	3 (3.5)	
TG/NB identity, female sex at birth	4 (4.3)	3 (3.5)	
Race/Ethnicity			0.003
White, non-Latinx/e	33 (35.1)	34 (40.0)	
Black, non-Latinx/e	42 (44.7)	22 (25.9)	
Latinx/e, any race	10 (10.6)	15 (17.6)	
Other race/multi-racial, non-Latinx/e	2 (2.1)	11 (12.9)	
Education			0.51
High school or less	25 (26.6)	21 (24.7)	
Some college	20 (21.3)	24 (28.2)	
College or higher	49 (52.1)	38 (44.7)	
Insurance status			0.26
Uninsured	8 (8.5)	13 (15.3)	
Public insurance	34 (36.2)	33 (38.8)	
Private insurance	48 (51.1)	35 (41.2)	
Source of HIVST kit			0.86
Online	73 (77.7)	64 (75.3)	
CBO or health center	14 (14.9)	13 (15.3)	
Someone they know	4 (4.3)	3 (3.5)	
Somewhere else	3 (3.2)	5 (3.9)	
MSM	25 (26.6)	23 (27.1)	1.00
Sex with a partner with HIV, lifetime	8 (12.3)	3 (5.1)	0.21
Sex with a partner with unknown HIV status, lifetime	62 (66.7)	63 (75.9)	0.19
IDU in past year	1 (1.1)	4 (4.7)	0.19
STI history			
None	55 (58.5)	48 (56.5)	0.88
STI > year ago	11 (11.7)	9 (10.6)	
STI in past year	23 (24.5)	24 (28.2)	
Condom use			0.61
Never	18 (19.1)	20 (23.5)	
Sometimes	63 (67.0)	54 (63.5)	
Always	12 (12.8)	8 (9.4)	
Unaware of PrEP at baseline survey	27 (28.7)	20 (23.5)	0.61
Intention to seek care, Scale 1–4 (SD)	3.22 (0.81)	2.96 (0.89)	0.057
Intentions to obtain PrEP, Scale 1–4 (SD)	2.34 (1.05)	2.24 (0.97)	0.53
Prior HIV test			0.61
No prior HIV test	24 (25.5)	18 (21.2)	
Last HIV test > 1 year ago	13 (13.8)	16 (18.8)	
HIV test in past year	57 (60.6)	51 (60.0)	

Rows may not sum to total N due to missing responses

*TG/NB identity* transgender/nonbinary identity, including gender diverse individuals; *CBO* community-based organization; *MSM* men who have sex with men (defined as assigned male sex at birth and prior anal sex with men); *IDU* injection drug use; *STI* sexually transmitted infection excluding HIV; *PrEP* pre-exposure prophylaxis; SD, standard deviation

a Calculated using Fisher’s exact test for categorical and binary variables, and t-test for continuous variables

**Table 3:** Factors correlated with seeing a provider and discussing PrEP

Factor	Saw a provider after self-testing		Discussed PrEP with provider	
	
	OR (95% CI)	AOR (95% CI)^a^	OR (95% CI)	AOR (95% CI)^b^
Age per year increase	1.03 (0.99, 1.07)	1.03 (0.98, 1.08)	0.96 (0.91, 1.03)	0.96 (0.90, 0.93)
Female sex at birth	1.01(0.55, 1.83)	1.00 (0.50, 2.00)	0.43 (0.16, 1.18)	0.30 (0.10, 0.93)*
Race/ethnicity				
White, non-Latinx/e	Reference	Reference	Reference	Reference
Black, non-Latinx/e	1.97 (0.97, 3.98)	2.50 (1.16, 5.41)*	2.27 (0.64, 8.08)	2.66 (0.77, 9.15)
Latinx/e, any race	0.69 (0.27, 1.75)	0.83 (0.29, 2.35)	4.83 (0.93, 25.18)	6.53 (1.23, 34.74)*
Other race/multi-racial, non-Latinx/e	0.19 (0.04, 0.91)*	0.09 (0.01, 0.74)*	7.26 (0.37, 142.67)	13.03 (0.89, 190.99)
Education				
High school or less	Reference	Reference	Reference	Reference
Some college	0.60 (0.26, 1.40)	0.56 (0.21, 1.47)	0.71 (0.19, 2.66)	1.24 (0.27, 5.80)
College or higher	0.95 (0.45, 2.00)	1.25 (0.45, 3.44)	0.35 (0.11, 1.14)	0.47 (0.13, 1.71)
Insurance				
Uninsured	Reference	Reference	Reference	Reference
Public insurance	1.67 (0.61, 4.57)	1.73 (0.60, 5.03)	0.92 (0.15, 5.56)	0.93 (0.16, 5.23)
Private insurance	2.22 (0.83, 5.97)	2.61 (0.91, 7.49)	0.69 (0.12, 4.05)	0.86 (0.17, 4.31)
Source of HIVST kit				
Online	Reference	Reference	Reference	Reference
CBO or health center	0.94 (0.41, 2.16)	1.15 (0.49, 2.69)	2.56 (0.73, 8.98)	3.33 (0.76, 14.57)
Someone they know	1.17 (0.25, 5.44)	1.55 (0.21, 11.53)	1.54 (0.15, 16.19)	7.34 (0.39, 139.35)
Somewhere else	0.53 (0.12, 2.30)	0.51 (0.057, 4.58)	2.31 (0.19, 27.77)	2.12 (0.31, 14.41)
MSM	0.91 (0.47, 1.78)	1.14 (0.38, 3.46)	5.24 (1.81, 15.14)*	18.37 (1.89, 178.33)*
Sex with a partner with HIV, lifetime	2.58 (0.65, 10.26)	3.90 (0.41, 36.9)	1.39 (0.24, 7.97)	3.90 (0.41, 36.95)
Sex with a partner with unknown HIV status, lifetime	0.68 (0.35, 1.32)	0.85 (0.40, 1.81)	0.40 (0.15, 1.12)	0.85 (0.40, 1.81)
IDU in past year	0.42 (0.07, 2.36)	0.49 (0.08, 2.86)	3.84 (0.23, 65.27)	0.49 (0.08, 2.86)
STI in past year	1.07 (0.42, 2.73)	0.78 (0.25, 2.49)	0.80 (0.16, 4.08)	0.78 (0.25, 2.49)
Condom use				
Never	Reference	Reference	Reference	Reference
Sometimes	1.30 (0.62, 2.70)	0.98 (0.40, 2.39)	0.87 (0.37, 2.04)	0.75 (0.20, 2.89)
Always	1.67 (0.55, 5.01)	1.57 (0.43, 5.78)	0.36 (0.66, 1.98)	0.48 (0.05, 5.14)
Aware of PrEP	0.80 (0.40, 1.59)	0.79 (0.36, 1.74)	0.92 (0.31, 2.74)	0.74 (0.20, 2.65)
Intentions to seek care^c^	1.44 (0.98, 2.10)	1.55 (0.96, 2.49)	1.59 (0.68, 3.74)	1.51 (0.93, 2.45)
Intentions to obtain PrEP^c^	1.12 (0.82, 1.53)	1.31 (0.88, 1.93)	4.29 (0.52, 35.39)	1.31 (0.88, 1.93)

* Denotes p-value < 0.05

a Each model adjusted for age, sex, race/ethnicity, and insurance

b Each model adjusted for age, sex, race/ethnicity

c Per unit increase on a 1–4 scale

## References

[1] Fauci AS, Redfield RR, Sigounas G, Weahkee MD, Giroir BP. Ending the HIV epidemic: a plan for the United States. JAMA. 2019;321(9):844–5. 10.1001/jama.2019.1343. 30730529

[2] Myers JE, Braunstein SL, Xia Q, Scanlin K, Edelstein Z, Harriman G, Redefining prevention and care: a status-neutral approach to HIV. Open Forum Infect Dis. 2018;5(6):ofy097. 10.1093/ofid/ofy097. 29977957 PMC6016418

[3] McNairy ML, El-Sadr WM. A paradigm shift: focus on the HIV prevention continuum. Clin Infect Dis. 2014;59 Suppl(Suppl 1):S12–5. 10.1093/cid/ciu251. 24926026 PMC4141493

[4] Sullivan PS, DuBose SN, Castel AD, Hoover KW, Juhasz M, Guest JL, Equity of PrEP uptake by race, ethnicity, sex and region in the United States in the first decade of PrEP: a population-based analysis. Lancet Reg Health Am. 2024;33:100738. 10.1016/j.lana.2024.100738. 38659491 PMC11041841

[5] Sullivan PS, Satcher Johnson A, Pembleton ES, Stephenson R, Justice AC, Althoff KN, Epidemiology of HIV in the USA: epidemic burden, inequities, contexts, and responses. The Lancet. 2021;397(10279):1095–106. 10.1016/s0140-6736(21)00395-0. PMC12707558 33617774

[6] Centers for Disease Control and Prevention. HIV surveillance supplemental report: Estimated HIV incidence and prevalence in the United States, 2018–2022. Report. 21/2024. Available from: https://stacks.cdc.gov/view/cdc/156513. Accessed 7 Dec 2024.

[7] US Food & Drug Administration. Important information on use of unapproved human immunodeficiency virus (HIV) blood sample self-collection kits. October 29, 2024. Available from: https://www.fda.gov/vaccines-blood-biologics/safety-availability-biologics/important-information-use-unapproved-human-immunodeficiency-virus-HIV-blood-sample-self-collection. Accessed 10 Jan 2025.

[8] MacGowan RJ, Chavez PR, Borkowf CB, Owen SM, Purcell DW, Mermin JH, Effect of internet-distributed HIV self-tests on HIV diagnosis and behavioral outcomes in men who have sex with men: a randomized clinical trial. JAMA Intern Med. 2020;180(1):117–25. 10.1001/jamainternmed.2019.5222. 31738378 PMC6865312

[9] Katz DA, Golden MR, Hughes JP, Farquhar C, Stekler JD. HIV self-testing increases HIV testing frequency in high-risk men who have sex with men: a randomized controlled trial. J Acquir Immune Defic Syndr. 2018;78(5):505–12. 10.1097/qai.0000000000001709. 29697595 PMC6037557

[10] Pathela P, Jamison K, Blank S, Daskalakis D, Hedberg T, Borges C. The HIV pre-exposure prophylaxis (PrEP) cascade at NYC Sexual Health Clinics: navigation is the key to uptake. J Acquir Immune Defic Syndr. 2020;83(4):357–64. 10.1097/qai.0000000000002274. 31904700

[11] Jin G, Shi H, Du J, Guo H, Yuan G, Yang H, Pre-exposure prophylaxis care continuum for HIV risk populations: an umbrella review of systematic reviews and meta-analyses. AIDS Patient Care STDS. 2023;37(12):583–615. 10.1089/apc.2023.0158. 38011347

[12] Hecht J, Sanchez T, Sullivan PS, DiNenno EA, Cramer N, Delaney KP. Increasing access to HIV testing through direct-to-consumer HIV self-test distribution—United States, March 31, 2020-March 30, 2021. MMWR Morb Mortal Wkly Rep. 2021;70(38):1322–5. 10.15585/mmwr.mm7038a2. 34555001 PMC8459896

[13] Zhang Y, Goh SM, Tapa J, Johnson CC, Chow EPF, Zhang L, Linkage to care and prevention after HIV self-testing: a systematic review and meta-analysis. J Int AIDS Soc. 2024;27(12):e26388. 10.1002/jia2.26388. 39663188 PMC11634545

[14] Bonett S, Dowshen N, Bauermeister J, Meanley S, Wirtz AL, Celentano DD, Characterizing the PrEP continuum for black and latinx sexual and gender minority youth. AIDS Behav. 2022;26(4):1211–21. 10.1007/s10461-021-03476-6. 34546472 PMC8934745

[15] Lightfoot MA, Campbell CK, Moss N, Treves-Kagan S, Agnew E, Kang Dufour MS, Using a social network strategy to distribute HIV self-test kits to African American and Latino MSM. J Acquir Immune Defic Syndr. 2018;79(1):38–45. 10.1097/qai.0000000000001726. 29771792

[16] Sharma A, Chavez PR, MacGowan RJ, McNaghten AD, Mustan-ski B, Gravens L, Willingness to distribute free rapid home HIV test kits and to test with social or sexual network associates among men who have sex with men in the United States. AIDS Care. 2017;29(12):1499–503. 10.1080/09540121.2017.1313386. 28393612 PMC12186660

[17] Sanchez T, MacGowan RJ, Hecht J, Keralis JM, Ackah-Toffey L, Bourbeau A, Findings from the first year of a federally funded, direct-to-consumer HIV self-test distribution program—United States, March 2023-March 2024. MMWR Morb Mortal Wkly Rep. 2024;73(24):558–64. 10.15585/mmwr.mm7324a4. 38900702 PMC11199019

[18] Philadelphia Department of Public Health Division of HIV Health. Surveillance report, 2022. December 28, 2023. Available from: https://www.phila.gov/media/20231228103020/HIVSurveillance_Report_2022.pdf. Accessed 20 Nov 2024.

[19] Centers for Disease Control and Prevention. National HIV behavioral surveillance (NHBS). February, 2024. Available from: https://www.cdc.gov/HIV-data/nhbs/index.html. Accessed 7 Dec 2024.

[20] Montaño DE, Kasprzyk D. Theory of reasoned action, theory of planned behavior, and the integrated behavioral model. In: Glanz K, Rimer BK, Viswanath KV, editors. Health behavior, theory, research, and practice. San Francisco/Hoboken: Jossey-Bass/Wiley; 2015. p. 95–124.

[21] Westreich D, Greenland S. The table 2 fallacy: presenting and interpreting confounder and modifier coefficients. Am J Epidemiol. 2013;177(4):292–8. 10.1093/aje/kws412. 23371353 PMC3626058

[22] Baugher AR, Trujillo L, Kanny D, Freeman JQ, Hickey T, Sionean C, Racial, ethnic, and gender disparities in awareness of preexposure prophylaxis among HIV-negative heterosexually active adults at increased risk for HIV infection—23 urban areas, United States, 2019. MMWR Morb Mortal Wkly Rep. 2021;70(47):1635–9. 10.15585/mmwr.mm7047a3. 34818317 PMC8612510

[23] Finlayson T, Cha S, Xia M, Trujillo L, Denson D, Prejean J, Changes in HIV preexposure prophylaxis awareness and use among men who have sex with men—20 urban areas, 2014 and 2017. MMWR Morb Mortal Wkly Rep. 2019;68(27):597–603. 10.15585/mmwr.mm6827a1. 31298662 PMC6741853

[24] Kanny D, Jeffries WLt, Chapin-Bardales J, Denning P, Cha S, Finlayson T, Racial/ethnic disparities in HIV preexposure prophylaxis among men who have sex with men—23 urban areas, 2017. MMWR Morb Mortal Wkly Rep. 2019;68(37):801–6. 10.15585/mmwr.mm6837a2. PMC6755820 31536484

[25] Seidman D, Logan R, Weber S, Gandhi A, Blackstock O. Rei-magining preexposure prophylaxis provision for women in the United States. Clin Infect Dis. 2022;74(12):2243–8. 10.1093/cid/ciab959. 35482659 PMC9890470

[26] Fishbein M. A reasoned action approach to health promotion. Med Decis Mak. 2008;28(6):834–44. 10.1177/0272989x08326092. PMC2603050 19015289

[27] Walsh JL. Applying the information-motivation-behavioral skills model to understand PrEP intentions and use among men who have sex with men. AIDS Behav. 2019;23(7):1904–16. 10.1007/s10461-018-2371-3. 30554396 PMC6571043

[28] Bien-Gund CH, Shaw PA, Agnew-Brune C, Baugher A, Brady KA, Gross R, HIV self-testing and risk behaviors among men who have sex with men in 23 US cities, 2017. JAMA Netw Open. 2022;5(12):e2247540–e. 10.1001/jamanetworkopen.2022.47540. 36534398 PMC9856873

[29] Anderson AN, Higgins CM, Haardörfer R, Holstad MM, Nguyen MLT, Waldrop-Valverde D. Disparities in retention in care among adults living with HIV/aids: a systematic review. AIDS Behav. 2020;24(4):985–97. 10.1007/s10461-019-02679-2. 31555931

[30] Aaron E, Blum C, Seidman D, Hoyt MJ, Simone J, Sullivan M, Optimizing delivery of HIV preexposure prophylaxis for women in the United States. AIDS Patient Care STDS. 2018;32(1):16–23. 10.1089/apc.2017.0201. 29323558 PMC5756936

[31] Irie WC, Blackstock OJ. A call for PrEP discussions with black women—be a gardener. JAMA Health Forum. 2024;5(5):e241130–e. 10.1001/jamahealthforum.2024.1130. 38787540

[32] Wray TB, Chan PA, Klausner JD, Ward LM, Ocean EMS, Carr DJ, The effects of regular home delivery of HIV self-testing and follow-up counselling on HIV testing and prevention outcomes in men who have sex with men who test infrequently in the United States: a pragmatic, virtual randomized controlled trial. J Int AIDS Soc. 2024;27(7):e26318. 10.1002/jia2.26318. 39020453 PMC11254576

[33] Johnson AL, Butts SA, Rodriguez E, Craker L, Kanamori M, Doblecki-Lewis S. “Test-to-PrEP”: assessing reach and adoption of a new approach to increase HIV testing and PrEP knowledge using HIV self-test kit distribution through PrEP clients’ social networks. J Acquir Immune Defic Syndr. 2023;94(5):421–8. 10.1097/qai.0000000000003294. 37949445 PMC10651164

[34] Blackstock OJ, Platt J, Golub SA, Anakaraonye AR, Norton BL, Walters SM, A pilot study to evaluate a novel pre-exposure prophylaxis peer outreach and navigation intervention for women at high risk for HIV infection. AIDS Behav. 2021;25(5):1411–22. 10.1007/s10461-020-02979-y. 32748159 PMC8284566

[35] Krakower DS, Gruber S, Hsu K, Menchaca JT, Maro JC, Kruskal BA, Development and validation of an automated HIV prediction algorithm to identify candidates for pre-exposure prophylaxis: a modelling study. Lancet HIV. 2019;6(10):e696–704. 10.1016/S2352-3018(19)30139-0. 31285182 PMC7522919

[36] Albernas A, Patel MD, Cook RL, Vaddiparti K, Prosperi M, Liu Y. HIV risk score and prediction model in the United States: a scoping review. AIDS Behav. 2025. 10.1007/s10461-025-04702-1. PMC12353520 40185966

