# Supplementary material for: HIV Prevention Continuum Outcomes Following Implementation of a Municipal HIV Self-Testing Program
Source: AIDS Behav. 2025 Aug 14;30(1):14–23. doi: 10.1007/s10461-025-04842-4 (PMC12573104; doi:10.1007/s10461-025-04842-4)
Supplement: Supplementary file 1 — Supplementary file1 (PDF 1306 kb) [file 10461_2025_4842_MOESM1_ESM.pdf]

TEST. LOVE. REPEAT.

PHILLY, KEEP ON TESTING!

WHAT DOES A **POSITIVE** RESULT MEAN?

If your test shows two lines you may be HIV positive.
As with any test, there is always the risk of false results. So it is important you inform your doctor or local health clinic immediately that your **OraQuick®** test showed a positive result. A healthcare professional will need to retest you to confirm those initial results. Until that retest, it is important to not engage in activities that could result in passing HIV to others.

If your follow up test does confirm you are HIV positive, and you live in Philadelphia, you can access care and medicines regardless of your insurance status, ability to pay or immigration or legal status. Call the **Health Information Helpline** at **215-985-2437**

WHAT DOES A **NEGATIVE** RESULT MEAN?

If your test shows one line you are likely HIV negative.
As with any test there is always the risk of false results. If you think you maybe have been exposed to HIV in the last three months, but your test is negative, it's important to test again in another three months because you could be in what's called the **"window period"** – a time you could have HIV, but tests cannot yet detect the virus.

If a follow-up test confirms the negative result, there are ways to ensure you stay that way - including PrEP and condoms.

Visit **PhillyKeepOnLoving.com** to learn more

Department of
Public Health

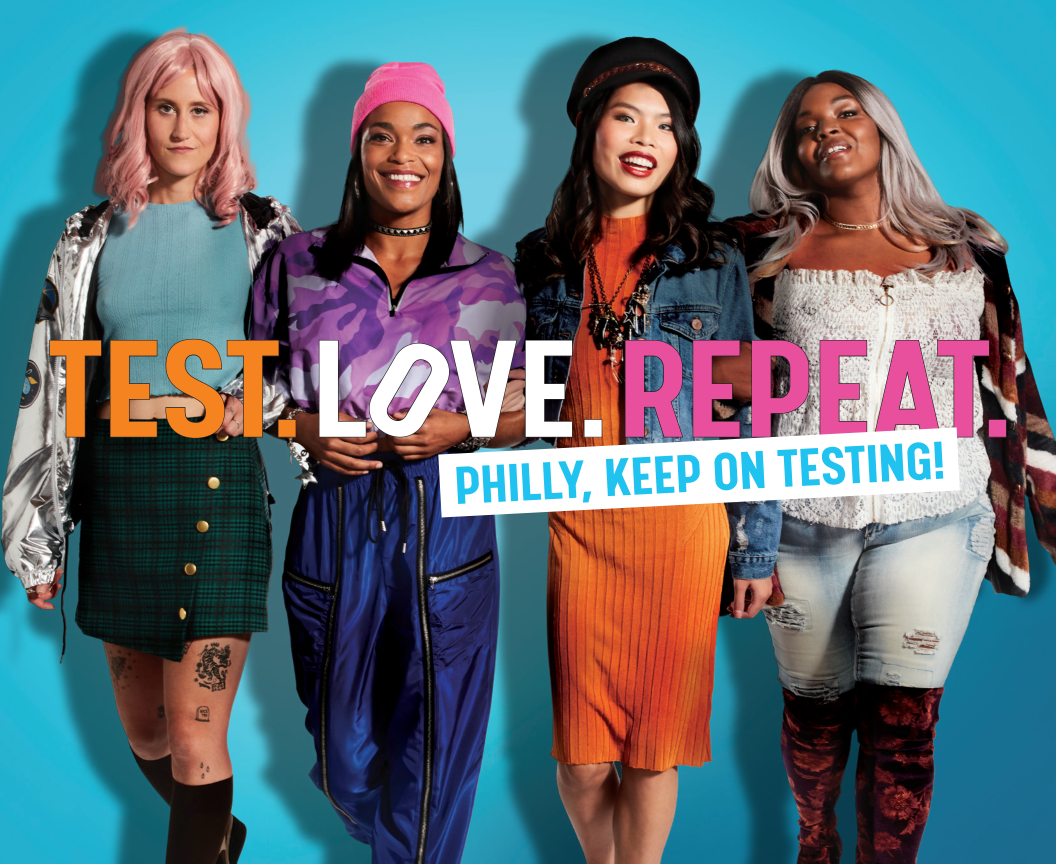
TEST. LOVE. REPEAT.
PHILLY, KEEP ON TESTING!

WHAT TO DO IF YOU TEST POSITIVE

Go to Health Center 1 **(1930 S. Broad Street)** or your doctor for a test to confirm your result.

Learning you're HIV positive can be overwhelming - but it doesn't have to be.

Today's treatment has changed what it means to live with HIV. You can expect to live a long, productive, healthy life that includes sex, relationships and family.

WHAT TO DO IF YOU'RE HIV NEGATIVE

Visit **phillykeeponloving.com** for information on PrEP and other HIV prevention options. PrEP is a daily pill that can keep your sex-life HIV free.

CALL FOR QUESTIONS

1.866.436.6527 OraQuick Support Center (24/7)

215.985.2437 (Health Information Helpline)

Weekdays **8am - 5:30pm**

Department of Public Health
