# Supplementary material for: HIV Prevention Continuum Outcomes Following Implementation of a Municipal HIV Self-Testing Program
Source: AIDS Behav. 2025 Aug 14;30(1):14–23. doi: 10.1007/s10461-025-04842-4 (PMC12573104; doi:10.1007/s10461-025-04842-4)
Supplement: Supplementary file 2 — Supplementary file2 (PDF 93 kb) [file 10461_2025_4842_MOESM2_ESM.pdf]

Initial Survey

Test Kit Information

Please enter the 6-digit code of the test kit you ordered.

____
(6-digit code)

Personal Contact Information

The following information is collected to compensate you for your participation.

Please enter a mobile phone number

____

Please enter an email address

____

1 Select your age in years

◯ 17
◯ 18
◯ 19
◯ 20
◯ 21
◯ 22
◯ 23
◯ 24
◯ 25
◯ 26
◯ 27
◯ 28
◯ 29
◯ 30
◯ 31
◯ 32
◯ 33
◯ 34
◯ 35
◯ 36
◯ 37
◯ 38
◯ 39
◯ 40
◯ 41
◯ 42
◯ 43
◯ 44
◯ 45
◯ 46
◯ 47
◯ 48
◯ 49
◯ 50
◯ 51
◯ 52
◯ 53
◯ 54
◯ 55
◯ 56
◯ 57
◯ 58
◯ 59
◯ 60
◯ 61
◯ 62
◯ 63
◯ 64
◯ 65
◯ 66
◯ 67
◯ 68
◯ 69
◯ 70
◯ 71
◯ 72
◯ 73
◯ 74
◯ 75
◯ 76
◯ 77
◯ 78
◯ 79
◯ 80
◯ 81
◯ 82
◯ 83
◯ 84
◯ 85

◯ 86
◯ 87
◯ 88
◯ 89
◯ 90
◯ 91
◯ 92
◯ 93
◯ 94
◯ 95
◯ 96
◯ 97
◯ 98
◯ 99
◯ 100

You indicated you are 17 years of age. In order to receive payment for this survey, please enter your parent or guardian's name.

Your parent or guardian will NOT be contacted.

____

Please enter your parent or guardian's date of birth.

____

Enter your date of birth

____

Enter your address (Line 1)

____

Enter your address (Line 2, e.g., Apartment number)

____

---

2 Enter your zip code

____
(5-digit zip code)

Socio-Demographic Information

3 What terms best express how you describe your gender identity?

- ◯ Woman
- ◯ Man
- ◯ Non-binary
- ◯ Transgender man/Female-to-male (FTM)
- ◯ Transgender woman/Male-to-female (MTF)
- ◯ Gender non-binary/Genderqueer/Gender nonconforming
- ◯ Agender
- ◯ Bigender
- ◯ None of these describe me
- ◯ Prefer not to answer

4 What was your sex assigned at birth on your birth certificate?

- ◯ Female
- ◯ Male
- ◯ Intersex
- ◯ None of these describe me
- ◯ Prefer not to answer

5 What is your race?

- ☐ American Indian or Alaska Native
- ☐ Black or African American
- ☐ Asian
- ☐ Native Hawaiian or Other Pacific Islander
- ☐ White
- ☐ Some other race: ______
- ☐ Prefer not to answer

(Mark one or more boxes.)

6 Are you of Hispanic, Latino, or Spanish origin?

- ◯ No, not of Hispanic, Latino, or Spanish origin
- ◯ Yes, of Hispanic, Latino, or Spanish origin
- ◯ Prefer not to answer

7 What is the highest level of education you have achieved outside or in the United States? Grades roughly equivalent to years of school.

- ◯ Have never gone to school
- ◯ 5th grade or less
- ◯ 6th to 8th grade
- ◯ 9th to 12th grade, no diploma
- ◯ High school graduate or GED completed
- ◯ Some college level / Technical / Vocational degree
- ◯ Bachelor's degree
- ◯ Other advanced degree (Master's, Doctoral degree)
- ◯ Prefer not to answer
- ◯ Don't know

8 What is the primary kind of health insurance or health care plan that you have now?

- ◯ I do NOT have health insurance
- ◯ Private (purchased directly or through Employment)
- ◯ Public (Medicare, Medicaid, Tricare)
- ◯ Don't know
- ◯ Prefer not to answer

9 In 2021, what was your total household income before taxes?

- ◯ Less than $15,000
- ◯ $15,000 - $19,999
- ◯ $20,000 - $24,999
- ◯ $25,000 - $34,999
- ◯ $35,000 - $49,999
- ◯ $50,000 - $74,999
- ◯ $75,000 - $99,999
- ◯ $100,000 and above
- ◯ Prefer not to answer

10	Which best describes your sexual orientation?	◯ Straight<br>◯ Asexual or demisexual<br>◯ Gay<br>◯ Bisexual<br>◯ Queer<br>◯ Pansexual<br>◯ Do not use label<br>◯ Other: ______<br>◯ Prefer not to say
11	How do you define your primary relationship status ?	◯ I am single<br>◯ I am dating casually<br>◯ I have a boyfriend or girlfriend<br>◯ I have a partner or lover<br>◯ My partner and I have had a commitment ceremony<br>◯ I am in a civil union or domestic partnership<br>◯ I am legally married<br>◯ Prefer not to say
12	How do you and your partner handle sex outside of your relationship?	◯ Neither of us has sex with others; we are monogamous<br>◯ Only I have sex with others<br>◯ Only they have sex with others<br>◯ Both of us have sex with others separately<br>◯ Both of us have sex with others together<br>◯ We both have sex with others separately and together<br>◯ I have sex with others, but don't know about my partner<br>◯ I don't have sex with others, but I don't know about my partner

HIV Testing

1	Have you ever had an HIV test before this one? Include any HIV test you have gotten in the past.	◯ Yes ◯ No ◯ Don't know
a)	In the past year, how many times have you tested for HIV?	__________________________
b)	When did you have your most recent HIV test before this one? Please give the approximate month and year.	__________________________<br>(MM/YYYY)
	The next set of questions ask about this HIV self-test.	
2	What was the result of your HIV self-test?	◯ Positive<br>◯ Negative<br>◯ Indeterminate<br>◯ Don't know<br>◯ Prefer not to answer
	You indicated you tested positive with the HIV self-test. Is this the first time you have tested positive?	◯ Yes<br>◯ No
	When did you first test positive for HIV? That is, when were you diagnosed with HIV?<br><br>If you don't know the exact date, an approximation is fine.	__________________________
	You indicated you don't know the result of your test. Have you taken the test yet?	◯ Yes<br>◯ No
3	How easy was it to use the test?	◯ Very difficult<br>◯ Somewhat difficult<br>◯ Neither difficult nor easy<br>◯ Somewhat easy<br>◯ Very easy
4	Where did you get this test?	◯ phillykeeponloving website<br>◯ Health center<br>◯ Someone I know gave it to me<br>◯ Somewhere else: ______
5	Was this the first time you have used a HIV self-test?	◯ Yes<br>◯ No<br>◯ Don't know<br>◯ Prefer not to answer
a)	Before this test, how many times have you used a self-test?	__________________________
b)	Before this test, when did you have your most recent HIV self-test? Please give the approximate month and year.	__________________________<br>(MM/YYYY)

6 If you were given an additional three HIV self-tests, what would you do with them?
For example, some people would save them for later. Other people would give them to their friends.

7 Please rate the following statements.

		Strongly disagree	Disagree	Neither disagree nor agree	Agree	Strongly agree	Prefer not to answer
1	I would feel uncomfortable going to a doctor's office or clinic for HIV testing.	◯	◯	◯	◯	◯	◯
2	I would prefer using HIV self-tests rather than going to a clinic to get tested.	◯	◯	◯	◯	◯	◯
3	It was easy to get an HIV self-test kit.	◯	◯	◯	◯	◯	◯
4	Self-testing meets my needs as a way of getting HIV tested.	◯	◯	◯	◯	◯	◯
5	Self-testing is appealing to me as a way of getting HIV tested.	◯	◯	◯	◯	◯	◯
6	Conducting the self-test was easy.	◯	◯	◯	◯	◯	◯
7	I would recommend HIV self-testing to a friend	◯	◯	◯	◯	◯	◯
8	I would recommend HIV self-testing to a partner	◯	◯	◯	◯	◯	◯

Sexual History

1	Have you ever had vaginal sex?	◯ Yes<br>◯ No<br>◯ Prefer not to answer
a)	When was the last time you had vaginal sex?	◯ In the past 3 months<br>◯ 3-6 months ago<br>◯ 6-12 months ago<br>◯ More than a year ago<br>◯ Prefer not to answer
2	Have you ever had anal sex with a man?	◯ Yes<br>◯ No<br>◯ Prefer not to answer
a)	When was the last time you had anal sex with a man?	◯ In the past 3 months<br>◯ 3-6 months ago<br>◯ 6-12 months ago<br>◯ More than a year ago<br>◯ Prefer not to answer
3	Have you had sex with someone whose HIV status you did not know?	◯ Yes<br>◯ No<br>◯ Don't know<br>◯ Prefer not to answer
4	Have you had sex with someone with HIV?	◯ Yes<br>◯ No<br>◯ Don't know<br>◯ Prefer not to answer
a)	When was the last time you had sex with someone with HIV?	◯ In the past 3 months<br>◯ 3-6 months ago<br>◯ 6-12 months ago<br>◯ More than a year ago<br>◯ Prefer not to answer
b)	Did that person(s) have an undetectable viral load?	◯ Yes, they had an undetectable viral load<br>◯ No, they had a detectable viral load<br>◯ I don't know if they had an undetectable viral load<br>◯ Prefer not to answer
5	Have you ever been diagnosed with a sexually transmitted infection (STI or STD)?<br><br>Examples of sexually transmitted infections include gonorrhea, chlamydia, syphilis, trichomoniasis	◯ Yes<br>◯ No<br>◯ Don't know<br>◯ Prefer not to answer
a)	When was the last time you were diagnosed with an STI or STD?	◯ In the past 3 months<br>◯ 3-6 months ago<br>◯ 6-12 months ago<br>◯ More than a year ago<br>◯ Prefer not to answer

6	About how often do you use a condom when having vaginal or anal sex?	◯ Never<br>◯ Sometimes<br>◯ Most of the time<br>◯ All the time<br>◯ Prefer not to answer
7	Have you ever given or gotten money or drugs for sex?	◯ Yes<br>◯ No<br>◯ Prefer not to answer
8	Have you ever injected drugs?	◯ Yes ◯ No ◯ Prefer not to answer
a)	Have you injected drugs in the past 12 months?	◯ Yes<br>◯ No<br>◯ Prefer not to answer
b)	Have you ever shared needles, syringes or other equipment to inject drugs?	◯ Yes<br>◯ No<br>◯ Prefer not to answer

Medical Care

1	Do you currently have a regular doctor or health care provider? This is also known as a primary care provider.	◯ Yes<br>◯ No<br>◯ Don't know<br>◯ Prefer not to answer
2	Have you gotten an HIV test from your primary care provider?	◯ Yes ◯ No ◯ Don't know
3	Have you discussed your sexual health with your primary care provider?	◯ Yes ◯ No ◯ Don't know
4	Have you discussed your sexual orientation with your primary care provider?	◯ Yes ◯ No ◯ Don't know
5	Have you discussed PrEP with your primary care provider?	◯ Yes ◯ No ◯ Don't know

PrEP use

1	Before today, have you heard of people taking anti-HIV medicines or an injection BEFORE sex or using drugs to lower the chance of getting HIV? This is called pre-exposure prophylaxis, or PrEP.	◯ Yes<br>◯ No<br>◯ Prefer not to answer
2	Before today, have you ever taken PrEP?	◯ Yes<br>◯ No<br>◯ Prefer not to answer
a)	About how many total months have you taken PrEP?	______________________<br>(Number of months)
b)	Are you taking PrEP right now?	◯ Yes<br>◯ No<br>◯ Prefer not to answer
c)	What kind of PrEP are you taking?	◯ I am taking injectable PrEP<br>◯ I am taking daily PrEP pills<br>◯ I am taking intermittent PrEP when I have sex ("event-driven")
c)	What were your reasons for stopping PrEP?	☐ Had bad side effects<br>☐ Worried about side effects of PrEP<br>☐ Unable to pay for PrEP (too expensive)<br>☐ Kept forgetting to take PrEP<br>☐ Unable to make it to PrEP medical visits<br>☐ Unable to afford PrEP medical visits<br>☐ No longer at risk for HIV<br>☐ Use other safe-sex strategies, specify: ______<br>☐ Other: ______<br>(Check all that apply.)
d)	When was the last time you took PrEP? Please give the approximate month and year.	______________________<br>(MM/YYYY)
3	Please indicate if you think the following statements are true or false:	
a)	One form of PrEP is a daily pill you can take to reduce your risk of becoming infected with HIV.	◯ True ◯ False ◯ Don't know
b)	Another form of PrEP is an injection that you can get every 8 weeks.	◯ True ◯ False ◯ Don't know
c)	PrEP use can lower the risk of HIV to almost 0.	◯ True ◯ False ◯ Don't know

Intentions to Seek Care and Use PrEP

During the next month, I will:

		Definitely will not do	Probably will not do	Probably will do	Definitely will do	Prefer not to answer
1	Talk to a health care provider about my sexual health	◯	◯	◯	◯	◯
2	Talk to a health care provider about HIV	◯	◯	◯	◯	◯
3	Talk to a health care provider about PrEP	◯	◯	◯	◯	◯
4	Seek out more information about PrEP	◯	◯	◯	◯	◯
5	Get a prescription for PrEP	◯	◯	◯	◯	◯

PrEP Linkage

We would like to improve the PrEP services that we can offer. One way we can help is to have someone help you get PrEP, make appointments, and provide advice about PrEP, called a PrEP navigator.

Please rate the following from strongly disagree to strongly agree:

		Strongly disagree	Disagree	Neither disagree nor agree	Agree	Strongly agree	Prefer not to answer
1	I would be more likely to get PrEP if someone could help me get an appointment with a PrEP provider.	○	○	○	○	○	○
2	I would accept a phone call or text from someone to help me get PrEP.	○	○	○	○	○	○

How important would it be that the PrEP navigator:

		Not important	Slightly important	Fairly important	Important	Very important	Prefer not to answer
3	Have taken PrEP	○	○	○	○	○	○
4	Is taking PrEP right now	○	○	○	○	○	○
5	Have formal medical training	○	○	○	○	○	○
6	Is similar to your age	○	○	○	○	○	○
7	Have a similar race or ethnicity	○	○	○	○	○	○
8	Have a similar culture	○	○	○	○	○	○
9	Have a similar sexual orientation	○	○	○	○	○	○
10	Have a similar income	○	○	○	○	○	○
11	Have a similar neighborhood	○	○	○	○	○	○

Attitudes Toward PrEP

Do you agree or disagree with the following statements:

		Strongly disagree	Disagree	Neither disagree nor agree	Agree	Strongly agree	Prefer not to answer
1	People in my community would be interested in learning more about PrEP	○	○	○	○	○	○
2	People in my community would consider taking PrEP	○	○	○	○	○	○
3	My friends would be interested in learning more about PrEP	○	○	○	○	○	○
4	My friends would consider taking PrEP	○	○	○	○	○	○
5	I know people in my community taking PrEP	○	○	○	○	○	○
6	I have friends who are taking PrEP	○	○	○	○	○	○
7	Someone taking PrEP should keep their pills hidden	○	○	○	○	○	○
8	People are judged because they take PrEP	○	○	○	○	○	○
9	I would have sex with someone who is taking PrEP	○	○	○	○	○	○
10	Taking PrEP makes people see you as slutty	○	○	○	○	○	○
11	Taking PrEP gets you praise (or respect) for being responsible	○	○	○	○	○	○
12	My friends would support me taking PrEP	○	○	○	○	○	○
13	Taking PrEP causes problems with your sex partner(s)	○	○	○	○	○	○
14	I would feel proud to take PrEP	○	○	○	○	○	○
15	People taking PrEP experience verbal harassment	○	○	○	○	○	○
16	People on PrEP are taking care of their health	○	○	○	○	○	○
17	My family would be supportive of me taking PrEP	○	○	○	○	○	○

How difficult would it be for you to:

		Very hard to do	Hard to do	Easy to do	Very easy to do	Prefer not to answer
1	Seek out more information about PrEP to decide if it is right for you?	○	○	○	○	○
2	Take a medicine like PrEP every day?	○	○	○	○	○
3	Get a PrEP injection every 8 weeks?	○	○	○	○	○
4	Visit in-person with a health provider every three months to get PrEP?	○	○	○	○	○
5	Visit remotely (over the telephone or video call, or telehealth appointment) with a health provider every three months to get PrEP?	○	○	○	○	○
6	Discuss PrEP with a friend?	○	○	○	○	○

Mental Health

The next few questions will ask you about your mental health. Over the last 2 weeks how often have you been bothered by the following problems?

		Not at all	Several days	More than half the days	Nearly every day
1	Little interest or pleasure in doing things	○	○	○	○
2	Feeling down, depressed or hopeless	○	○	○	○
3	Trouble falling or staying asleep, or sleeping too much	○	○	○	○
4	Feeling tired or having little energy	○	○	○	○
5	Poor appetite or overeating	○	○	○	○
6	Feeling bad about yourself - or that you are a failure or have let yourself or your family down	○	○	○	○
7	Trouble concentrating on things, such as reading the newspaper or watching television	○	○	○	○
8	Moving or speaking so slowly that other people could have noticed. Or the opposite - being so fidgety or restless that you have been moving around a lot more than usual	○	○	○	○
10	Feeling nervous, anxious or on edge	○	○	○	○
11	Not being able to stop or control worrying	○	○	○	○
12	Worrying too much about different things	○	○	○	○
13	Trouble relaxing	○	○	○	○
14	Being so restless that it's hard to sit still	○	○	○	○
15	Becoming easily annoyed or irritable	○	○	○	○
16	Feeling afraid as if something awful might happen	○	○	○	○

Substance Use

1	How often did you have a drink containing alcohol in the past year?	◯ Never<br>◯ Monthly or less<br>◯ Two to four times a month<br>◯ Two to three times a week<br>◯ Four or more times a week
a)	During the past year, how many drinks did you have on a typical day that you consumed alcohol?	◯ 1 or 2<br>◯ 3 or 4<br>◯ 5 or 6<br>◯ 7 to 9<br>◯ 10 or more
2	How often did you have six or more drinks on one occasion in the past year?	◯ Never<br>◯ Less than monthly<br>◯ Monthly<br>◯ Weekly<br>◯ Daily or almost daily
3	Have you ever used cocaine or methamphetamines in any form?	◯ Yes ◯ No
a)	Have you used cocaine or methamphetamines in the past 6 months?	◯ Yes ◯ No
4	Have you ever used substances to enhance your sexual performance or pleasure?	◯ Yes ◯ No
a)	Were these substances prescribed by a clinician?	◯ Yes ◯ No
5	Have you ever used heroin or other opiates?	◯ Yes ◯ No
a)	Have you used heroin or other opiates in the past 6 months?	◯ Yes ◯ No

Interest in TelePrEP

The Philadelphia Department of Public Health is going to offer PrEP through telehealth, or TelePrEP. With TelePrEP, you can see a provider with your smartphones or computer, without going to a clinic. You would do fingerstick blood tests on yourself to test for HIV and mail the tests (or have the option to go to a nearby lab to get your HIV test). PrEP would then be mailed to you.

1 During the past 12 months, have you had an appointment with a doctor, nurse, or other health professional by video or phone? ◯ Yes ◯ No ◯ Don't know

2 Would you be interested in obtaining PrEP through telehealth services? ◯ Yes ◯ No ◯ Don't know

Please rate the following statements:

		Strongly disagree	Disagree	Neither disagree nor agree	Agree	Strongly agree
4	I would feel uncomfortable going to a doctor's office or clinic for PrEP.	◯	◯	◯	◯	◯
5	I would prefer getting PrEP from a virtual provider (telehealth) instead of going in-person.	◯	◯	◯	◯	◯
6	I would be more likely to get PrEP if I could get a long acting injection every 8 weeks instead of a daily pill.	◯	◯	◯	◯	◯

7 Would you like us to contact you about obtaining PrEP services? ◯ Yes ◯ No

8 Would you like us to contact you about obtaining additional sexual health care? ◯ Yes ◯ No

9 What is your preferred mode of contact?
◯ Phone Call
◯ Text message (SMS)
◯ E-mail
