# Supplementary figures and images for: HIV Prevention Continuum Outcomes Following Implementation of a Municipal HIV Self-Testing Program
Source: AIDS Behav. 2025 Aug 14;30(1):14–23. doi: 10.1007/s10461-025-04842-4 (PMC12573104; doi:10.1007/s10461-025-04842-4)

**Supplemental Figure 3: Adapted Integrated Behavioral Model for PrEP initiation**

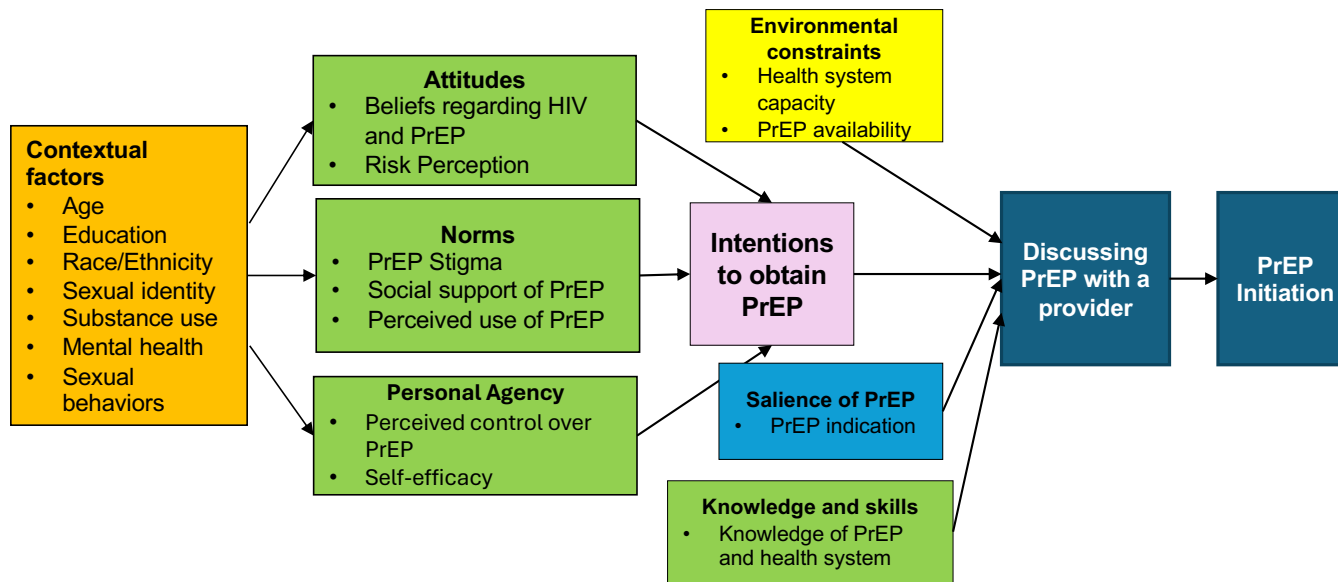

Supplement: Supplementary file 3 — Supplementary file3 (PDF 37 kb) [file 10461_2025_4842_MOESM3_ESM.pdf]
