# Supplementary material for: HIV Prevention Continuum Outcomes Following Implementation of a Municipal HIV Self-Testing Program
Source: AIDS Behav. 2025 Aug 14;30(1):14–23. doi: 10.1007/s10461-025-04842-4 (PMC12573104; doi:10.1007/s10461-025-04842-4)
Supplement: Supplementary file 4 — Supplementary file4 (PDF 41 kb) [file 10461_2025_4842_MOESM4_ESM.pdf]

Follow-Up Survey

Please complete the survey below.

Thank you!

	How likely are you to use another HIV self-test in the next 6 months?	◯ Very unlikely<br>◯ Unlikely<br>◯ Likely<br>◯ Very likely<br>◯ Prefer not to answer
	In the past month, have you recommended HIV self-testing to a friend?	◯ Yes<br>◯ No<br>◯ Prefer not to answer
	In the past month, have you recommended HIV self-testing to a partner?	◯ Yes<br>◯ No<br>◯ Prefer not to answer
1	In the past month, have you used another HIV self-test?	◯ Yes<br>◯ No<br>◯ Prefer not to answer
2	In the past month, have you gotten an HIV test in a clinic or health center?	◯ Yes<br>◯ No<br>◯ Prefer not to answer
3	In the past month, have you seen a health care provider in-person?	◯ Yes, I have seen a provider in-person<br>◯ No<br>◯ Prefer not to answer
4	In the past month, have you seen a health care provider virtually?	◯ Yes, I have seen a provider virtually<br>◯ No<br>◯ Prefer not to answer
a)	Since getting your HIV self-test, have you made an appointment to see a health care provider?	◯ Yes ◯ No ◯ Prefer not to answer
	What are the reasons you have not made an appointment?	☐ Not able to find a provider<br>☐ No time to see a provider<br>☐ Worried about cost<br>☐ No insurance<br>☐ Not interested or no need<br>☐ Other: ______<br>☐ Prefer not to answer<br>(Select all that apply.)
5	Did you talk with your health care provider about sexual health?	◯ Yes ◯ No ◯ Prefer not to answer
6	Have you been tested for any sexually transmitted infections (STI or STD) in the past month?	◯ Yes ◯ No ◯ Prefer not to answer
7	Have you been diagnosed with an STI or STD in the past month?	◯ Yes ◯ No ◯ Prefer not to answer

8	Did you talk with your health care provider about PrEP?	◯ Yes ◯ No ◯ Prefer not to answer
9	Are you taking PrEP right now?	◯ Yes ◯ No ◯ Prefer not to answer
a)	What method of PrEP are you currently taking?	◯ I am taking a pill for PrEP<br>◯ I am getting a PrEP injection<br>◯ Prefer not to answer
b)	When did you start taking PrEP? Please give the approximate month and year.	____________________<br>(MM/YYYY)

Intentions to Seek Care and Use PrEP

Please respond to the following questions based on what you intend to do during the next month.

		Definitely will not do	Probably will not do	Probably will do	Definitely will do	Prefer not to answer
1	Talk to a health care provider about my sexual health	◯	◯	◯	◯	◯
2	Talk to a health care provider about HIV	◯	◯	◯	◯	◯
3	Talk to a health care provider about PrEP	◯	◯	◯	◯	◯
4	Seek out more information about PrEP	◯	◯	◯	◯	◯
5	Get a prescription for PrEP	◯	◯	◯	◯	◯
